# 5-(Pyridin-2-yl)-3,3′-bi(1*H*-1,2,4-triazole)

**DOI:** 10.1107/S1600536811027449

**Published:** 2011-07-13

**Authors:** Zhouqing Xu, Xinping Zhao, Qiang Wang

**Affiliations:** a The Department of Physics–Chemistry, Henan Polytechnic University, Jiao Zuo, 454000, People’s Republic of China; b The Hospital of Henan Polytechnic University, Jiao Zuo, 454000, People’s Republic of China

## Abstract

In the title mol­ecule, C_9_H_7_N_7_, the two triazole rings are twisted by an angle of 3.8 (5)°; the central triazole ring is twisted by 32.3 (6)° with respect to the pyridyl ring. The crystal packing consists of layers generated by inter­molecular N—H⋯N hydrogen bonds.

## Related literature

For related structures, see: Mai *et al.* (2009 ▶); Zhang *et al.* (2010 ▶). For the synthesis, see: Potts (1960 ▶); Wiley & Hart (1953 ▶).
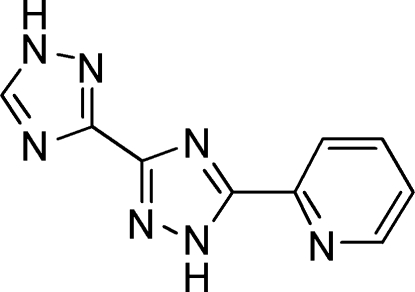


## Experimental

### 

#### Crystal data

C_9_H_7_N_7_
*M* *_r_* = 213.22
Monoclinic, 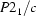

*a* = 12.372 (3) Å
*b* = 7.5361 (15) Å
*c* = 10.007 (2) Å
β = 93.670 (4)°
*V* = 931.1 (3) Å^3^
*Z* = 4
Mo *K*α radiation
μ = 0.11 mm^−1^
*T* = 296 K
0.24 × 0.20 × 0.20 mm

#### Data collection

Bruker SMART APEX diffractometer
Absorption correction: multi-scan (*SADABS*; Sheldrick, 1996 ▶) *T*_min_ = 0.975, *T*_max_ = 0.979
5150 measured reflections
1832 independent reflections
1072 reflections with *I* > 2σ(*I*)
*R*_int_ = 0.055

#### Refinement

*R*[*F* ^2^ > 2σ(*F* ^2^)] = 0.050
*wR*(*F* ^2^) = 0.156
*S* = 0.94
1832 reflections
154 parameters
H atoms treated by a mixture of independent and constrained refinement
Δρ_max_ = 0.26 e Å^−3^
Δρ_min_ = −0.22 e Å^−3^

### 

Data collection: *APEX2* (Bruker, 2003 ▶); cell refinement: *SAINT* (Bruker, 2003 ▶); data reduction: *SAINT*; program(s) used to solve structure: *SHELXS97* (Sheldrick, 2008 ▶); program(s) used to refine structure: *SHELXL97* (Sheldrick, 2008 ▶); molecular graphics: *SHELXTL* (Sheldrick, 2008 ▶); software used to prepare material for publication: *SHELXTL*.

## Supplementary Material

Crystal structure: contains datablock(s) I, global. DOI: 10.1107/S1600536811027449/ng5195sup1.cif

Structure factors: contains datablock(s) I. DOI: 10.1107/S1600536811027449/ng5195Isup2.hkl

Supplementary material file. DOI: 10.1107/S1600536811027449/ng5195Isup3.cml

Additional supplementary materials:  crystallographic information; 3D view; checkCIF report

## Figures and Tables

**Table 1:** Hydrogen-bond geometry (Å, °)

*D*—H⋯*A*	*D*—H	H⋯*A*	*D*⋯*A*	*D*—H⋯*A*
N6—H1⋯N4^i^	0.98 (4)	1.93 (4)	2.891 (3)	165 (3)
N2—H2⋯N3^ii^	0.99 (3)	1.90 (4)	2.878 (3)	167 (3)

Symmetry codes: (i) 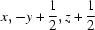; (ii) 
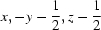
.

**Fig. 1.**
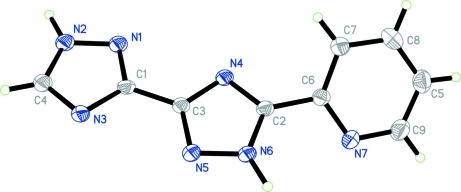
The molecular structure of the title molecule, with the atom-numbering scheme. Displacement ellipsoids are drawn at the 30% probability level.

**Fig. 2.**
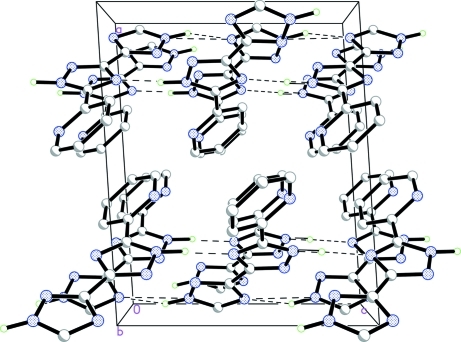
A partial packing diagram of the title compound. Hydrogen bonds are shown as dashed lines, viewed down the b axis.

## supplementary crystallographic information

### Comment

During the past decads, copounds containling trazole subunits have been intensively studied due to their diverse biological activities, such as fungicide, herbicide, medicine, *etc*., and have become a central focus in the sudy of agricultural, medicinal and material chemicals. Furthermore, the study on crystal structures and properties of the new metal-organic frameworks (MOFs) of N-containing ligands have attracted considerable attentions during the past years for the their potential applications in polymeric materials, catalytic materials, biological materials optical materials and so on (Zhang, *et al.*, 2010). Therefore, in search for new multidentate ligands, we have synthesized the title compound and determined its structure.

The molecule structure of title compound was shown in the Fig.1, the lengths and angles are within normal ranges. In triazol ring, the average C—N bond length is 1.336 (5) Å, which is shorter than C—N (mean 1.461 (2) Å) (Zhang, *et al.*, 2010), but longer than C=N (mean 1.269 (3) Å) (Mai, *et al.*, 2009). This is caused probably by electron delocalization in heterocyclic systems. In the crystal structure, the two triazole rings are almost coplanar, they are twisted by an angle of 3.8 (5)°. the central triazole ring form dihedral angles of 32.3 (6)° with the pyridyl ring. The crystal packing (Fig. 2) consists of two-dimensional infinite plane along the *b* axis generated by intermolecular interactions of N—H···N hydrogen bonds.

### Experimental

5-(Pyridin-2-yl)-1H,1'H-3,3'-bi(1,2,4-triazole)was prepared according to Wiley & Hart (1953) and Potts *et al.* (1960). The crystals suitable for crystallographic analysis were grown by recrystallization from DMF and ethanol solution as colorless block.

### Refinement

N-bound H-atoms were located in a difference map and refined freely. C-bound H atoms were positioned geometrically (C—H = 0.94 Å) and were constrained in a riding motion approximation with *U*_iso_(H) = 1.2*U*_eq_(C).

### Crystal data

**Table:**

C_9_H_7_N_7_	*F*(000) = 440
*M**_r_* = 213.22	*D*_x_ = 1.521 Mg m^−^^3^
Monoclinic, *P*2_1_/*c*	Mo *K*α radiation, λ = 0.71073 Å
*a* = 12.372 (3) Å	Cell parameters from 6322 reflections
*b* = 7.5361 (15) Å	θ = 2.0–27.9°
*c* = 10.007 (2) Å	µ = 0.11 mm^−^^1^
β = 93.670 (4)°	*T* = 296 K
*V* = 931.1 (3) Å^3^	Block, colourless
*Z* = 4	0.24 × 0.20 × 0.20 mm

### Data collection

**Table:**

Bruker SMART APEX diffractometer	1832 independent reflections
Radiation source: fine-focus sealed tube	1072 reflections with *I* > 2σ(*I*)
graphite	*R*_int_ = 0.055
ω scan	θ_max_ = 26.0°, θ_min_ = 1.7°
Absorption correction: multi-scan (*SADABS*; Sheldrick, 1996)	*h* = −15→12
*T*_min_ = 0.975, *T*_max_ = 0.979	*k* = −9→9
5150 measured reflections	*l* = −12→12

### Refinement

**Table:**

Refinement on *F*^2^	Secondary atom site location: difference Fourier map
Least-squares matrix: full	Hydrogen site location: inferred from neighbouring sites
*R*[*F*^2^ > 2σ(*F*^2^)] = 0.050	H atoms treated by a mixture of independent and constrained refinement
*wR*(*F*^2^) = 0.156	*w* = 1/[σ^2^(*F*_o_^2^) + (0.0843*P*)^2^] where *P* = (*F*_o_^2^ + 2*F*_c_^2^)/3
*S* = 0.94	(Δ/σ)_max_ < 0.001
1832 reflections	Δρ_max_ = 0.26 e Å^−^^3^
154 parameters	Δρ_min_ = −0.22 e Å^−^^3^
0 restraints	Extinction correction: *SHELXL*, Fc^*^=kFc[1+0.001xFc^2^λ^3^/sin(2θ)]^-1/4^
Primary atom site location: structure-invariant direct methods	Extinction coefficient: 0.018 (5)

### Special details

**Table:**

Geometry. All e.s.d.'s (except the e.s.d. in the dihedral angle between two l.s. planes) are estimated using the full covariance matrix. The cell e.s.d.'s are taken into account individually in the estimation of e.s.d.'s in distances, angles and torsion angles; correlations between e.s.d.'s in cell parameters are only used when they are defined by crystal symmetry. An approximate (isotropic) treatment of cell e.s.d.'s is used for estimating e.s.d.'s involving l.s. planes.
Refinement. Refinement of *F*^2^ against ALL reflections. The weighted *R*-factor *wR* and goodness of fit *S* are based on *F*^2^, conventional *R*-factors *R* are based on *F*, with *F* set to zero for negative *F*^2^. The threshold expression of *F*^2^ > σ(*F*^2^) is used only for calculating *R*-factors(gt) *etc*. and is not relevant to the choice of reflections for refinement. *R*-factors based on *F*^2^ are statistically about twice as large as those based on *F*, and *R*- factors based on ALL data will be even larger.

### Fractional atomic coordinates and isotropic or equivalent isotropic displacement parameters (Å^2^)

**Table:**

	*x*	*y*	*z*	*U*_iso_*/*U*_eq_	
N4	0.22980 (16)	0.2080 (3)	0.46400 (19)	0.0342 (6)	
N6	0.23958 (18)	0.2161 (3)	0.6818 (2)	0.0370 (6)	
N2	0.07056 (18)	−0.2247 (3)	0.2744 (2)	0.0394 (6)	
N3	0.06963 (16)	−0.2067 (3)	0.4914 (2)	0.0367 (6)	
N5	0.18243 (17)	0.0672 (3)	0.6502 (2)	0.0372 (6)	
C1	0.1252 (2)	−0.0720 (3)	0.4381 (2)	0.0311 (6)	
N1	0.12802 (17)	−0.0763 (3)	0.3067 (2)	0.0387 (6)	
C2	0.26642 (19)	0.2993 (3)	0.5708 (2)	0.0329 (6)	
C3	0.1792 (2)	0.0682 (3)	0.5182 (2)	0.0324 (6)	
C4	0.0376 (2)	−0.2994 (3)	0.3838 (3)	0.0388 (7)	
H4	−0.0028	−0.4034	0.3851	0.047*	
N7	0.40073 (18)	0.4877 (3)	0.6744 (2)	0.0446 (6)	
C5	0.4476 (2)	0.7649 (4)	0.5774 (3)	0.0543 (8)	
H5	0.4900	0.8669	0.5828	0.065*	
C6	0.3285 (2)	0.4650 (3)	0.5701 (2)	0.0343 (6)	
C7	0.3123 (2)	0.5856 (3)	0.4680 (3)	0.0417 (7)	
H7	0.2620	0.5636	0.3969	0.050*	
C8	0.3724 (2)	0.7401 (4)	0.4729 (3)	0.0531 (8)	
H8	0.3618	0.8257	0.4064	0.064*	
C9	0.4592 (2)	0.6366 (4)	0.6738 (3)	0.0547 (8)	
H9	0.5113	0.6543	0.7437	0.066*	
H1	0.249 (2)	0.252 (4)	0.776 (4)	0.088 (11)*	
H2	0.062 (2)	−0.262 (4)	0.179 (3)	0.081 (11)*	

### Atomic displacement parameters (Å^2^)

**Table:**

	*U*^11^	*U*^22^	*U*^33^	*U*^12^	*U*^13^	*U*^23^
N4	0.0456 (13)	0.0338 (12)	0.0232 (11)	0.0004 (10)	0.0025 (9)	−0.0019 (9)
N6	0.0522 (14)	0.0363 (13)	0.0222 (12)	−0.0021 (11)	0.0009 (10)	−0.0032 (10)
N2	0.0552 (15)	0.0364 (13)	0.0262 (13)	0.0000 (11)	−0.0009 (10)	−0.0045 (10)
N3	0.0461 (13)	0.0346 (13)	0.0291 (12)	−0.0022 (10)	−0.0010 (10)	0.0007 (9)
N5	0.0483 (14)	0.0354 (13)	0.0276 (12)	−0.0030 (10)	−0.0001 (10)	−0.0029 (9)
C1	0.0411 (15)	0.0302 (14)	0.0220 (13)	0.0052 (11)	0.0015 (11)	0.0005 (11)
N1	0.0539 (15)	0.0371 (13)	0.0248 (12)	−0.0014 (11)	0.0011 (10)	−0.0023 (9)
C2	0.0402 (15)	0.0325 (15)	0.0257 (14)	0.0036 (12)	0.0003 (11)	−0.0006 (11)
C3	0.0422 (15)	0.0304 (14)	0.0246 (13)	0.0019 (11)	0.0022 (11)	−0.0009 (11)
C4	0.0485 (17)	0.0348 (15)	0.0329 (15)	0.0001 (12)	−0.0002 (12)	−0.0018 (12)
N7	0.0514 (14)	0.0434 (14)	0.0379 (14)	−0.0093 (12)	−0.0042 (11)	−0.0014 (11)
C5	0.061 (2)	0.0467 (19)	0.056 (2)	−0.0133 (15)	0.0099 (16)	−0.0040 (16)
C6	0.0399 (15)	0.0334 (15)	0.0299 (14)	0.0039 (12)	0.0041 (11)	−0.0020 (11)
C7	0.0461 (17)	0.0409 (16)	0.0380 (16)	0.0001 (13)	0.0017 (12)	0.0001 (13)
C8	0.057 (2)	0.0426 (17)	0.060 (2)	0.0022 (15)	0.0098 (16)	0.0114 (16)
C9	0.057 (2)	0.058 (2)	0.0487 (19)	−0.0165 (16)	−0.0029 (15)	−0.0037 (16)

### Geometric parameters (Å, °)

**Table:**

N4—C2	1.326 (3)	C2—C6	1.467 (3)
N4—C3	1.356 (3)	C4—H4	0.9300
N6—C2	1.336 (3)	N7—C9	1.336 (3)
N6—N5	1.353 (3)	N7—C6	1.341 (3)
N6—H1	0.98 (4)	C5—C9	1.366 (4)
N2—C4	1.319 (3)	C5—C8	1.368 (4)
N2—N1	1.353 (3)	C5—H5	0.9300
N2—H2	0.99 (3)	C6—C7	1.372 (4)
N3—C4	1.322 (3)	C7—C8	1.380 (4)
N3—C1	1.354 (3)	C7—H7	0.9300
N5—C3	1.319 (3)	C8—H8	0.9300
C1—N1	1.318 (3)	C9—H9	0.9300
C1—C3	1.461 (3)		
			
C2—N4—C3	102.9 (2)	N2—C4—N3	111.0 (2)
C2—N6—N5	110.4 (2)	N2—C4—H4	124.5
C2—N6—H1	131 (2)	N3—C4—H4	124.5
N5—N6—H1	119 (2)	C9—N7—C6	115.9 (2)
C4—N2—N1	109.8 (2)	C9—C5—C8	118.6 (3)
C4—N2—H2	131.2 (19)	C9—C5—H5	120.7
N1—N2—H2	118.9 (19)	C8—C5—H5	120.7
C4—N3—C1	102.0 (2)	N7—C6—C7	123.6 (2)
C3—N5—N6	102.2 (2)	N7—C6—C2	115.3 (2)
N1—C1—N3	114.9 (2)	C7—C6—C2	121.1 (2)
N1—C1—C3	121.6 (2)	C6—C7—C8	118.7 (3)
N3—C1—C3	123.5 (2)	C6—C7—H7	120.7
C1—N1—N2	102.3 (2)	C8—C7—H7	120.7
N4—C2—N6	109.7 (2)	C5—C8—C7	118.7 (3)
N4—C2—C6	126.1 (2)	C5—C8—H8	120.6
N6—C2—C6	124.2 (2)	C7—C8—H8	120.6
N5—C3—N4	114.8 (2)	N7—C9—C5	124.5 (3)
N5—C3—C1	121.9 (2)	N7—C9—H9	117.8
N4—C3—C1	123.3 (2)	C5—C9—H9	117.8
			
C2—N6—N5—C3	0.7 (3)	N1—C1—C3—N4	4.1 (4)
C4—N3—C1—N1	0.6 (3)	N3—C1—C3—N4	−176.4 (2)
C4—N3—C1—C3	−179.1 (2)	N1—N2—C4—N3	0.5 (3)
N3—C1—N1—N2	−0.3 (3)	C1—N3—C4—N2	−0.6 (3)
C3—C1—N1—N2	179.4 (2)	C9—N7—C6—C7	−0.9 (4)
C4—N2—N1—C1	−0.2 (3)	C9—N7—C6—C2	178.9 (2)
C3—N4—C2—N6	0.6 (3)	N4—C2—C6—N7	−147.8 (3)
C3—N4—C2—C6	179.8 (2)	N6—C2—C6—N7	31.2 (3)
N5—N6—C2—N4	−0.9 (3)	N4—C2—C6—C7	32.0 (4)
N5—N6—C2—C6	180.0 (2)	N6—C2—C6—C7	−149.0 (2)
N6—N5—C3—N4	−0.3 (3)	N7—C6—C7—C8	−0.8 (4)
N6—N5—C3—C1	179.4 (2)	C2—C6—C7—C8	179.5 (2)
C2—N4—C3—N5	−0.2 (3)	C9—C5—C8—C7	−0.9 (5)
C2—N4—C3—C1	−179.9 (2)	C6—C7—C8—C5	1.7 (4)
N1—C1—C3—N5	−175.6 (2)	C6—N7—C9—C5	1.7 (4)
N3—C1—C3—N5	3.9 (4)	C8—C5—C9—N7	−0.9 (5)

### Hydrogen-bond geometry (Å, °)

**Table:**

*D*—H···*A*	*D*—H	H···*A*	*D*···*A*	*D*—H···*A*
N6—H1···N4^i^	0.98 (4)	1.93 (4)	2.891 (3)	165 (3)
N2—H2···N3^ii^	0.99 (3)	1.90 (4)	2.878 (3)	167 (3)

Symmetry codes: (i) *x*, −*y*+1/2, *z*+1/2; (ii) *x*, −*y*−1/2, *z*−1/2.

## References

[bb1] Bruker (2003). *APEX2* and *SAINT* Bruker AXS Inc., Madison, Wisconsin, USA.

[bb2] Mai, X., Xia, H.-Y., Cao, Y.-S., Lu, X.-S. & Liao, Y.-J. (2009). *Z. Kristallogr. New Cryst. Struct.* **224**, 547–548.

[bb3] Potts, K. T. (1960). *Chem. Rev.* **61**, 87–127.

[bb4] Sheldrick, G. M. (1996). *SADABS* University of Göttingen, Germany.

[bb5] Sheldrick, G. M. (2008). *Acta Cryst.* A**64**, 112–122. 10.1107/S0108767307043930 18156677

[bb6] Wiley, R. H. & Hart, A. J. (1953). *J. Org. Chem.* **18**, 1368–1371.

[bb7] Zhang, C.-H., Zhang, J.-J., Li, W. & Liu, B.-H. (2010). *Z. Kristallogr. New Cryst. Struct.* **225**, 599–600.

